# Paradoxical heat sensation as a manifestation of thermal hypesthesia: a study of 1090 patients with lesions of the somatosensory system

**DOI:** 10.1097/j.pain.0000000000003014

**Published:** 2023-08-11

**Authors:** Jan Vollert, Francesca Fardo, Nadine Attal, Ralf Baron, Didier Bouhassira, Elena K. Enax-Krumova, Rainer Freynhagen, Per Hansson, Troels S. Jensen, Dilara Kersebaum, Christoph Maier, Esther Pogatzki-Zahn, Andrew S.C. Rice, Juliane Sachau, Ellen L. Schaldemose, Märta Segerdahl, Manon Sendel, Thomas R. Tölle, Nanna B. Finnerup, Rolf-Detlef Treede

**Affiliations:** a Pain Research, MSk Lab, Department of Surgery and Cancer, Imperial College, London, United Kingdom; b Division of Neurological Pain Research and Therapy, Department of Neurology, University Hospital Schleswig-Holstein, Campus Kiel, Germany; c Department of Anaesthesiology, Intensive Care and Pain Medicine, University Hospital Muenster, Münster, Germany; d Department of Neurophysiology, Mannheim Center for Translational Neuroscience MCTN, Medical Faculty Mannheim, Ruprecht Karls University, Heidelberg, Germany; e Center of Functionally Integrative Neuroscience, Department of Clinical Medicine, Aarhus University, Aarhus, Denmark; f Danish Pain Research Center, Department of Clinical Medicine, Aarhus University, Aarhus, Denmark; g INSERM U-987, Centre d'Evaluation et de Traitement de la Douleur, CHU Ambroise Paré, Boulogne-Billancourt, France, Université Versailles-Saint-Quentin, Versailles, France; h Department of Neurology, BG University Hospital Bergmannsheil gGmbH, Ruhr-University Bochum, Bochum, Germany; i Department of Anaesthesiology, Critical Care Medicine, Pain Therapy and Palliative Care, Pain Center Lake Starnberg, Benedictus Hospital, Tutzing, Germany; j Department of Anaesthesiology, Klinikum Rechts der Isar, Technische Universität München, Munich, Germany; k Department of Pain Management and Research, Division of Emergencies and Critical Care, Oslo University Hospital, Oslo, Norway; l Department of Molecular Medicine and Surgery, Karolinska Institutet, Stockholm, Sweden; n University Hospital of Pediatrics and Adolescent Medicine, Ruhr-University Bochum, Bochum, Germany; o Department of Neurobiology, Care Sciences and Society, Karolinska Institutet, Stockholm, Sweden; p MS Medical Consulting, Stockholm, Sweden; q Department of Neurology, Klinikum Rechts der Isar, Technische Universität München, Munich, Germany; m Department of Neurology, Aarhus University Hospital, Aarhus, Denmark

**Keywords:** Paradoxical heat sensation, Quantitative sensory testing, Neuropathic pain, Neuropathy, Neuropathic Pain Symptom Inventory

## Abstract

Paradoxical heat sensation (PHS) is the perception of warmth when the skin is cooled. Paradoxical heat sensation rarely occurs in healthy individuals but more frequently in patients suffering from lesions or disease of the peripheral or central nervous system. To further understand mechanisms and epidemiology of PHS, we evaluated the occurrence of PHS in relation to disease aetiology, pain levels, quantitative sensory testing parameters, and Neuropathic Pain Symptom Inventory (NPSI) items in patients with nervous system lesions. Data of 1090 patients, including NPSI scores from 404 patients, were included in the analysis. We tested 11 quantitative sensory testing parameters for thermal and mechanical detection and pain thresholds, and 10 NPSI items in a multivariate generalised linear model with PHS, aetiology, and pain (yes or no) as fixed effects. In total, 30% of the neuropathic patients reported PHS in contrast to 2% of healthy individuals. The frequency of PHS was not linked to the presence or intensity of pain. Paradoxical heat sensation was more frequent in patients living with polyneuropathy compared with central or unilateral peripheral nerve lesions. Patients who reported PHS demonstrated significantly lower sensitivity to thermal perception, with lower sensitivity to normally painful heat and cold stimuli. Neuropathic Pain Symptom Inventory scores were lower for burning and electric shock–like pain quality for patients with PHS. Our findings suggest that PHS is associated with loss of small thermosensory fibre function normally involved in cold and warm perception. Clinically, presence of PHS could help screening for loss of small fibre function as it is straightforward to measure or self-reported by patients.

## Abstract 2

Sensory profiles of 1090 patients reveal loss of small thermosensory fibre function to be associated with paradoxical heat sensation.

## 1. Introduction

Paradoxical heat sensation (PHS) occurs when cooling of the skin is perceived as warmth instead of cold. Typically, single cold stimuli are perceived accurately, whereas PHS is experienced during temporal alternation of warming and cooling on the same area of the skin. Paradoxical heat sensation is rare in healthy individuals but can be provoked by preheating to noxious temperatures,^13^ application of topical capsaicin, or by high-frequency stimulation.^27,35^ In patients, PHS frequently occurs in individuals suffering from diverse neurological disorders of both peripheral and central origin.^14,19,21,23,28,29,40^ Although generally described as a painless sensation, some studies have reported that PHS can also be perceived as painful or burning.^14^

Altered sensation such as PHS can be evoked in healthy individuals by applying nerve compression blocks. Classic studies showed that when the conduction of myelinated fibres is blocked by ischaemic or nerve compression, pain induced by single cold stimuli is perceived at higher temperatures and is typically described as burning hot.^11,38,39^ These results suggest that the paradoxical sensation of heat and burning elicited by cold stimuli is mediated by unmyelinated fibres (ie, C fibres) that are not affected by the compression. This interpretation is consistent with conduction velocity findings demonstrating that PHS is peripherally transmitted through C fibres.^30^ Mechanistic hypotheses have suggested that PHS is mediated through a specific class of C fibres, known as C2, normally responsive to cooling, heating, and menthol in humans^6^ or by disinhibition of the heat–pinch–cold (HPC) pathway in the peripheral or central nervous system.^6,14^ The HPC disinhibition model is a common explanation of perceptual changes with high similarity to PHS, such as the thermal grill illusion or A-fibre block.^7,11,38,39^

However, these human experimental models are generally linked to a painful, burning heat, rather than painless warmth as mostly reported for PHS as perceived by patients. In this article, we analysed PHS in relation to aetiology (the specific injury or disease of the somatosensory system), presence or absence of pain, somatosensory profiles, descriptors of spontaneous pain, and abnormal sensations in 1090 patients with painful or painless polyneuropathy, unilateral peripheral nerve damage, or central lesions. Patients were part of the prospectively collected (N) European database and published previously in part.^2,19^

The objective of this study was to gain a better understanding of the clinical relevance of PHS in neuropathy by evaluating the relationship between paradoxical heat sensations, aetiology, presence or absence of pain, somatosensory profile, and pain symptoms. Furthermore, we aimed to explore putative mechanisms of this paradoxical phenomenon.

First, we compared the frequency of PHS across patient populations. Second, we tested whether an increase in PHS is associated with changes in the somatosensory profile, especially regarding sensory loss of cold and warm detection, combined with sensory gain of cold pain thresholds, equating PHS to the paradoxical sensations induced by an experimental A-fibre block.^11^ Finally, we analysed whether PHS could be related to specific neuropathic pain symptoms as captured by the Neuropathic Pain Symptom Inventory.

## 2. Materials and methods

### 2.1. Standard protocol approvals, registrations, and patient consents

The study was approved by the local ethics committees at each participating centre. All patients have given written informed consent for their data to be collected and analysed in the database. All procedures were conducted in accordance with the Declaration of Helsinki.

### 2.2. Study design

All patients were tested in a single setting, using the QST protocol of the German Research Network on Neuropathic Pain (DFNS),^26^ during which presence or absence of paradoxical heat sensation is recorded. Patients filled in the questionnaire before assessment. Fifteen pain facilities across Europe participated in gathering data in 3 consortia (German Research Network on Neuropathic Pain (DFNS), IMI (Innovative Medicines Initiative) EUROPAIN, and NEUROPAIN). All centres underwent a strict quality assessment.^18,37^ Data were collected in a central database in Bochum, Germany. An analysis of heterogeneity between the participating centres showed a high degree of homogeneity between the different centres, making it possible to analyse the database as a homogenous group.^33^ Subsets of this database as well as inclusion and exclusion have been published previously.^2,8,10,12,19,22,34,36^

### 2.3. Inclusion or exclusion criteria

We included patients with confirmed peripheral neuropathic pain^9,31^ due to polyneuropathy or unilateral peripheral lesions (peripheral nerve injury, postherpetic neuralgia, or radiculopathy); patients with central neuropathic pain due to spinal cord injury, syringomyelia, or stroke; and patients with similar painless conditions.^10,19^ Exclusion criteria were age < 18 years, insufficient language skills or other communication problems, pain treatment by topical local anaesthetics for ≥7 days in the last 4 months or by topical capsaicin in the last 6 months, comorbidities treated by anticonvulsants or antidepressants, other pain locations with pain intensities ≥6/10 on ≥15 days/month, spinal canal stenosis, peripheral vascular disease (Fontaine stage II or higher), pending litigation, and major cognitive or psychiatric disorders. Patients who were unable to detect any temperature change during thermal sensory limen (TSL) due to extensive sensory loss to thermal stimuli were excluded because PHS could not be determined (see below). We also excluded patients with unilateral syndromes who were also affected by contralateral neuropathy or painful conditions of the contralateral limb, as well as patients with incomplete records (eg, no precise diagnosis available, more than 2 missing variables of the QST in the affected area, and no information about age, sex, or other demographic data).

### 2.4. Quantitative sensory testing protocol and paradoxical heat sensation assessment

We used the QST protocol established by the DFNS.^25,26^ Patients suffering from polyneuropathy were tested on the dorsum of the foot, whereas patients suffering from other aetiologies were tested on the most affected area. In addition to PHS, QST according to the DFNS protocol assesses 12 other parameters: cold and warm detection thresholds (CDT and WDT), thermal sensory limen, cold and heat pain thresholds (CPT and HPT), mechanical pain threshold and sensitivity (MPT and MPS), dynamic mechanical allodynia (DMA), pressure pain threshold (PPT), wind-up ratio (WUR), tactile (mechanical) detection threshold (MDT), and vibration detection threshold.^25^ Thermal detection and pain thresholds were measured using either a TSA 2001-II (MEDOC, Israel) or a MSA (SOMEDIC SALES AB, Sweden) thermode at a rate of temperature change of 1°C/second.^25^ The number of PHS (if any) was recorded during assessment of the TSL. In this task, patients were instructed that the testing device would either cool or warm their skin several times, and they should press a stop button every time they felt a new temperature change. Furthermore, patients were instructed to describe the quality of the temperature change (eg, cold, warm, hot, or painfully hot) after each button press. This procedure consisted of alternated warming and cooling of the skin for a total of 6 consecutive temperature changes. The TSL procedure always started with warming of the skin, but no preheating to noxious temperatures was applied.^13^ The number of PHSs (ie, report of a warm sensation while the skin was cooled) varied between 0 and 3. According to the DFNS normative data, if patients were female and older than 50 years or male and older than 40 years and they were tested on the feet, abnormal frequency of PHS is defined as at least 2 PHSs during the 3 cooling temperature changes. In younger ages, any number of PHSs was considered abnormal. Henceforth, we will use the term “no PHS” to refer to “no elevated reports of PHS compared with normative data.” Patients who were unable to detect any temperature change during TSL were excluded because PHS could not be determined.

An initial assessment of data from 180 healthy individuals in the DFNS reference database revealed that all parameters, except PHS and DMA, were normally distributed or could be transformed to a standard normal distribution using a log transformation.^18,24,25^ Quantitative sensory testing raw or log-transformed results were normalised using a z-transformation, based on normative material defined for 5 different age decades, sex, and 4 testing sites (Magerl et al., 2010). If the patient's most prominent pain was in an upper limb, the values were z-transformed using the reference data for hands; if the most painful area was at a lower limb, the values were z-transformed using the reference data for feet. Assessments at the torso were z-transformed using the reference data for trunk, whereas all assessments at the face were z-transformed using the reference values for the cheek. Vibration detection threshold was compared with normative data assessed at the ulnar styloid for hand and arm, malleolus for foot and leg, costal arch for trunk, and zygomatic process for face. As a consequence of normalization, all measures had a mean = 0 and a standard deviation = 1 and were comparable between patients irrespective of their demographics or affected body area (eg, face or feet). Abnormal values were defined as values beyond the 95% confidence interval, corresponding to z values <−1.96 or >1.96.

### 2.5. The Neuropathic Pain Symptom Inventory questionnaire

The NPSI was developed to identify different dimensions of neuropathic pain.^5^ It comprises 10 items, including 5 descriptors of ongoing pain quality (burning, squeezing, or electric shocks), 3 descriptors of evoked pain (pain evoked by light touch, thermal stimuli, or light pressure or cold), and 2 reports of tingling and pins and needles. For each of the 10 items, patients rated the intensity on an 11-point rating scale, ranging from “zero or no [...]” to “10 or worst [...] imaginable.”

### 2.6. Statistics

We applied unpaired *t*-tests (2-tailed, *P* < 0.05) to investigate whether PHS could discriminate differences between the groups of patients with different aetiologies or with and without pain. Using multivariate analyses, we tested 11 normally distributed QST parameters and 10 NPSI items, with PHS (elevated or normal), aetiology (polyneuropathy, unilateral peripheral, or central), duration of disease (less than a year, 1 to 5 years, over 5 years), and presence or absence of pain as fixed main effects, and PHS × aetiology, PHS × duration, and PHS × pain as interaction effects. To avoid overinterpretation of false positive results due to alpha-cumulation and multiple testing, we applied a Benjamini–Hochberg correction to control the false discovery rate and only discuss effects that are significant models on a corrected *P* < 0.05 level.

### 2.7. Data access and availability

All data and materials are in the database hosted by the German Research Network of Neuropathic Pain (DFNS) and accessible to the first author (J.V.). Data access for purpose of replication can be requested through submitting an informal data request to the DFNS and will be granted by the DFNS board after review.

## 3. Results

### 3.1. Study population

A total of 1090 patients from 15 European centres were included in the analysis (polyneuropathy: 438 painful and 61 painless, unilateral peripheral nerve injury: 411 painful and 40 painless, and CNS lesion: 119 painful and 21 painless). The NPSI was not collected in all centres, and only for patients with painful conditions, and therefore was available from 404 patients only (polyneuropathy: 165, unilateral peripheral: 173, and CNS lesion: 66). Patients' demographics are presented in Table 1. Patients with elevated PHS were on average slightly older (59 years vs 57 years, *P* < 0.001), within patients with unilateral peripheral lesion more likely to appear after a radicular lesion, and within patients with central lesion less likely to appear after a stroke. There were no differences between painful and nonpainful conditions and neither in sex, duration of disease, or aetiology of polyneuropathy.

**Table 1:** Patient demographics.

	All patients	PHS	No PHS	*P*
n (%)	1090	330 (30%*)	760 (70%*)	
Sex (n [%] female)	523 (48%†)	149 (45%†)	374 (49%†)	0.22‡
Pain (n [%] chronic pain)	968 (89%†)	291 (88%†)	677 (89%†)	0.67‡
Age (mean ± standard deviation)	56.9 ± 14	59.4 ± 14	56.7 ± 15	<0.01§
Duration of disease (n [%])				0.12‡
Up to 1 year	235 (22%)	65 (20%†)	170 (22%†)	
1-5 years	491 (45%)	138 (42%†)	353 (46%†)	
Over 5 years	207 (19%)	70 (21%†)	137 (18%†)	
Unknown	157 (14%)	57 (17%†)	100 (13%†)	
Aetiology				<0.01‡
Polyneuropathy (n [%])	499 (46%)	208 (42%*)	291 (58%*)	0.44‡
Idiopathic	362 (73%)	153 (74%†)	209 (72%†)	
Diabetic	75 (15%)	31 (15%†)	44 (15%†)	
Chemotherapy induced	23 (5%)	8 (4%†)	15 (5%†)	
Toxic	12 (2%)	6 (3%†)	6 (2%†)	
HIV	12 (2%)	2 (1%†)	10 (3%†)	
Other	15 (3%)	8 (4%†)	7 (2%†)	
Unilateral peripheral lesions/diseases (n [%])	451 (41%)	89 (20%*)	362 (80%*)	<0.01‡
Peripheral nerve injury	259 (57%)	43 (48%†)	216 (60%†)	
Postherpetic neuralgia	102 (23%)	16 (18%†)	86 (24%†)	
Radicular lesion	90 (20%)	30 (34%†)	60 (17%†)	
Central lesions/diseases (n [%])	140 (13%)	33 (24%*)	107 (76%*)	0.01‡
Spinal cord injury	10 (7%)	5 (15%†)	5 (5%†)	
Syringomyelia	51 (36%)	4 (12%†)	47 (44%†)	
Stroke	79 (56%)	24 (73%†)	55 (51%†)	

* Percentage of patients with or without elevated PHS referring to all patients (horizontal).

† percentage of patients within given subgroup (vertical).

‡ *P* value derived from the chi^2^ test.

§ *P* value derived from the *t* test.

PHS, paradoxical heat sensation.

### 3.2. Frequency of paradoxical heat sensation

Abnormal PHS was reported by 30% of all included patients. Notably, although the percentage was higher in patients with loss of thermal detection (35%) compared with those with preserved thermal detection (29%), in absolute numbers, most patients with PHS had preserved thermal detection (n = 248) compared with those with loss of thermal detection (n = 82). This result is significantly different from the frequency of PHS in the DFNS reference data for healthy participants,^25^ which found that only 2% of healthy participants reported PHS in lower limbs of older participants (*P* < 0.001). Paradoxical heat sensation was more frequent in polyneuropathy (42%) in comparison to unilateral peripheral nervous lesions (20%) and central nervous lesions (24%) (*P* < 0.001), whereas there was no difference between unilateral peripheral and central nervous lesions (*P* = 0.33). Regarding painful vs painless conditions, we found no differences in the frequency of PHS, neither overall (*P* = 0.67) nor within aetiologies (polyneuropathy: *P* = 0.69, unilateral peripheral nervous lesions: *P* = 0.38, and central nervous lesions: *P* = 0.98).

### 3.3. Paradoxical heat sensation in relation to sensory profiles

**Figure 1.**
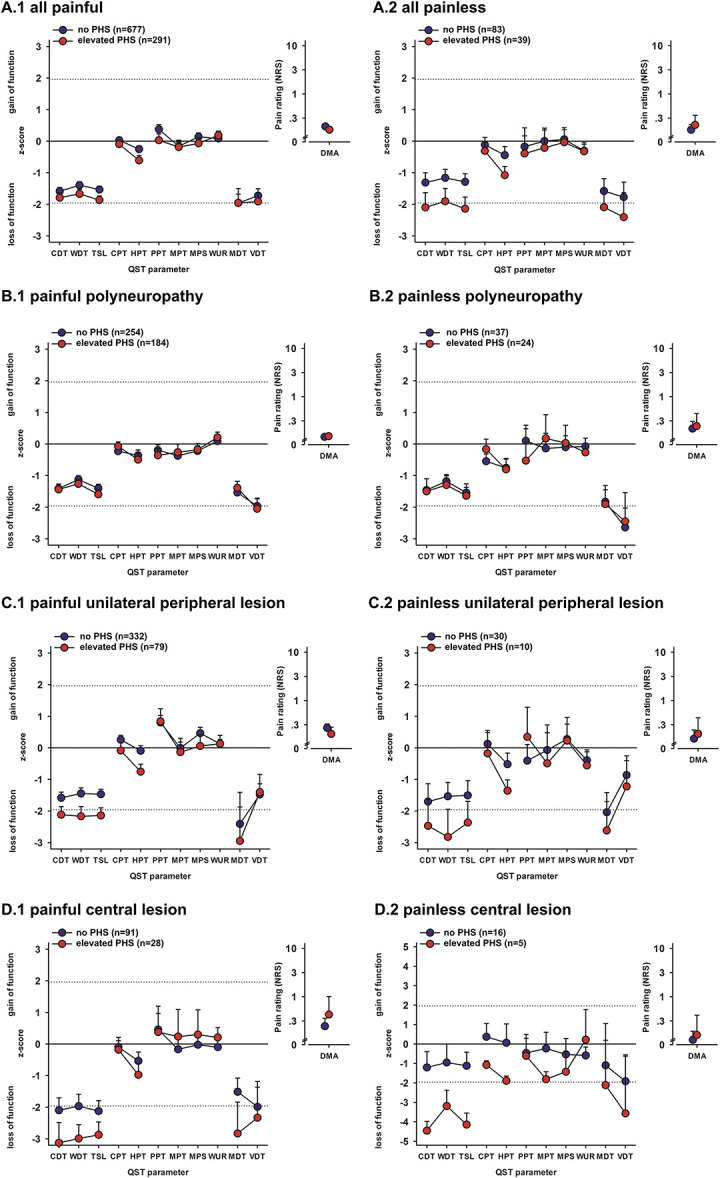
Quantitative sensory testing z profile of patients with elevated frequency of paradoxical heat sensation (PHS) in comparison to those without PHS for painful (1) and painless (2) lesion or disease of the somatosensory system. Z values between −1.96 and +1.96 indicate the 95% confidence interval of values found in healthy subjects. (A) all patients, (B) polyneuropathy, (C) unilateral peripheral nerve injury, and (D) central lesion. All values presented as mean ± 95% CI of the mean.

Figure 1 displays the sensory profile for all patient groups in relation to PHS, and all results of the multivariate analysis can be found in Table 2. In models corrected for aetiology, duration of disease, and presence or absence of pain, PHS had a significant effect on all thermal QST parameters, both thermal detection (CDT, WDT, and TSL) and pain thresholds (CPT and HPT). There was a significant interaction of PHS and aetiology for each of the thermal QST parameters, indicating that thermal thresholds were elevated in the presence of PHS for patients with unilateral nerve lesions and central lesions but not polyneuropathy. In addition, there was a significant interaction effect of PHS and presence of pain for thermal detection thresholds and MPT, indicating more loss of function associated with PHS in patients without pain. Finally, there was a significant interaction between PHS and duration of disease for CPT, PPT, and MPS. In the presence of PHS, more loss of function for CPT was detected. This was, however, not the case in patients with a disease duration of over 5 years. In patients with a disease duration of under 1 year there was a gain of function for PPT and MPS in the presence of PHS. With longer disease duration, the opposite (ie, a loss of function for PPT and MPS) could be shown.

**Table 2:** Multivariate analysis.

	Model	PHS	Aetiology	PHS × Aetiology	Duration	PHS × Duration	Pain	PHS*Pain
QST parameter								
CDT	**6.54 (0.00)**	**26.57 (0.00)**	**11.61 (0.00)**	**6.64 (0.00)**	0.55 (0.65)	0.14 (0.93)	0.16 (0.69)	**5.79 (0.02)**
WDT	**8.51 (0.00)**	**34.06 (0.00)**	**16.99 (0.00)**	**9.25 (0.00)**	0.56 (0.64)	0.05 (0.99)	0.59 (0.44)	**7.20 (0.01)**
TSL	**7.50 (0.00)**	**31.49 (0.00)**	**7.53 (0.00)**	**6.60 (0.00)**	0.29 (0.83)	0.78 (0.51)	0.00 (1.00)	**6.74 (0.01)**
CPT	**5.17 (0.00)**	**7.08 (0.01)**	1.62 (0.20)	**6.77 (0.00)**	0.75 (0.52)	**3.16 (0.02)**	0.50 (0.48)	2.80 (0.09)
HPT	**3.60 (0.00)**	**13.62 (0.00)**	0.26 (0.77)	**3.58 (0.03)**	0.19 (0.90)	2.13 (0.09)	0.85 (0.36)	2.69 (0.10)
PPT	**7.42 (0.00)**	0.00 (0.99)	**3.34 (0.04)**	0.67 (0.51)	**4.28 (0.01)**	**3.38 (0.02)**	3.46 (0.06)	0.12 (0.73)
MPT	**2.12 (0.02)**	0.68 (0.41)	0.06 (0.94)	0.79 (0.45)	**3.07 (0.03)**	2.12 (0.10)	1.40 (0.24)	**4.17 (0.04)**
MPS	**2.85 (0.00)**	0.01 (0.94)	1.14 (0.32)	0.25 (0.78)	3.34 (0.02)	**3.99 (0.01)**	0.09 (0.77)	0.00 (0.98)
WUR	**1.89 (0.04)**	0.81 (0.37)	0.69 (0.50)	0.83 (0.44)	0.22 (0.88)	1.60 (0.19)	**4.79 (0.03)**	0.00 (0.97)
MDT	1.77 (0.06)	2.46 (0.12)	1.58 (0.21)	1.09 (0.34)	0.59 (0.62)	1.04 (0.38)	0.19 (0.66)	0.71 (0.40)
VDT	**3.09 (0.00)**	1.45 (0.23)	**8.17 (0.00)**	0.49 (0.61)	0.92 (0.43)	0.56 (0.64)	0.34 (0.56)	0.13 (0.72)
**NPSI item**								
Burning	**2.46 (0.03)**	**4.54 (0.03)**	**3.13 (0.04)**	0.99 (0.37)	0.02 (1.00)	**2.99 (0.03)**		
Squeezing	1.19 (0.31)	0.00 (0.97)	1.91 (0.15)	1.55 (0.21)	1.43 (0.23)	0.46 (0.71)		
Pressure	1.98 (0.08)	1.71 (0.19)	**3.06 (0.05)**	0.03 (0.97)	2.09 (0.10)	0.68 (0.56)		
Electric shocks	**2.65 (0.02)**	**7.91 (0.01)**	0.39 (0.68)	1.09 (0.34)	2.20 (0.09)	1.24 (0.29)		
Stabbing	1.83 (0.11)	**4.05 (0.04)**	1.35 (0.26)	0.18 (0.84)	0.54 (0.66)	1.58 (0.19)		
Provoked by brushing	**7.28 (0.00)**	1.69 (0.19)	**11.17 (0.00)**	1.02 (0.36)	**4.62 (0.00)**	0.18 (0.91)		
Provoked by pressure	**8.97 (0.00)**	0.53 (0.47)	**13.24 (0.00)**	1.49 (0.23)	0.25 (0.86)	0.60 (0.61)		
Provoked by cold	**3.60 (0.00)**	0.15 (0.70)	**5.61 (0.00)**	2.73 (0.07)	0.88 (0.45)	0.60 (0.61)		
Pins and needles	**3.66 (0.00)**	1.58 (0.21)	**6.78 (0.00)**	2.29 (0.10)	0.93 (0.42)	0.37 (0.77)		
Tingling	**2.34 (0.04)**	2.66 (0.10)	1.85 (0.16)	2.29 (0.10)	0.90 (0.44)	0.59 (0.62)		

F value (*P* value). Bold values indicate *p* < 0.05.

Degrees of freedom: 7 (QST models) or 5 (NPSI models).

n (QST models), 1090; n (NPSI models), 404.

Pain is not included in NPSI models as only patients with chronic pain filled out the NPSI questionnaire

CDT, cold detection threshold; CPT, cold pain threshold; HPT, heat pain threshold; MDT, mechanical detection threshold; MPS, mechanical pain sensitivity; MPT, mechanical pain threshold; NPSI, Neuropathic Pain Symptom Inventory; PHS, paradoxical heat sensation; PPT, pressure pain threshold; QST, quantitative sensory testing; TSL, thermal sensory limen; VDT, vibration detection threshold; WDT, warm detection threshold; WUR = wind-up ratio.

### 3.4. Paradoxical heat sensation in relation to Neuropathic Pain Symptom Inventory items

**Figure 2.**
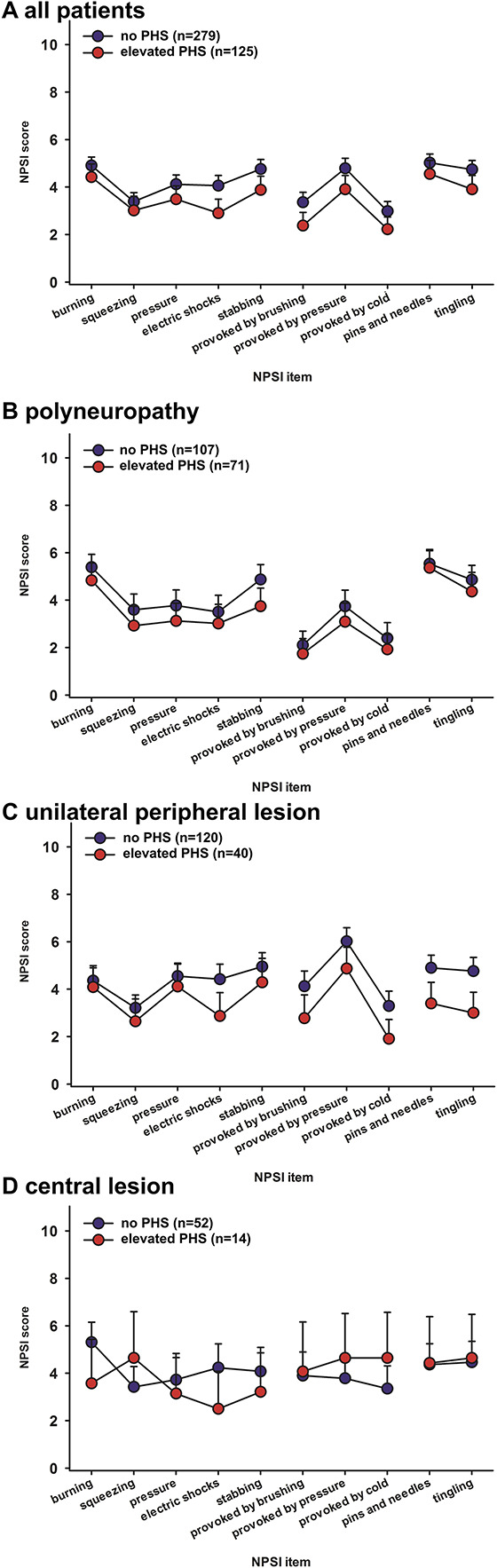
Neuropathic Pain Symptom Inventory profile of patients with elevated frequency and without paradoxical heat sensation. (A) all patients with neuropathic pain, (B) painful polyneuropathy, (C) unilateral peripheral neuropathic pain, and (D) central neuropathic pain. All values presented as mean ± 95% CI of the mean.

Neuropathic Pain Symptom Inventory profiles of all patient etiologies can be found in Figure 2. In the multivariate NPSI models, there was a significant effect of PHS on burning pain and electric shock–like pain, both being less frequent in patients with PHS. In addition, there was a significant interaction between PHS and duration of disease for burning pain, indicating more burning sensation for patients with presence of PHS and a disease duration below 1 year, and less burning sensation for patients with elevated PHS and a duration over a year.

## 4. Discussion

In this large clinical data set of patients experiencing peripheral and central nervous system diseases with and without neuropathic pain, abnormal PHS was frequently reported by all patient groups irrespective of whether they suffered from painful or painless lesion or disease of the somatosensory system (30% each). We found that PHS was much more frequent in patients with polyneuropathy (39%-42%) compared with unilateral peripheral or central lesions. Paradoxical heat sensation was associated with reduced thermosensitive small fibre function. The presence of PHS was not related to mechanical detection or pain thresholds, suggesting that PHS is not a sign of a general, but rather a thermosensory-specific small fibre dysfunction. Finally, we found an association between the presence of abnormal PHS and lower NPSI scores for electric shock–like and burning pain quality.

### 4.1. Paradoxical heat sensation is more frequent in polyneuropathy

Paradoxical heat sensation was significantly more frequent in polyneuropathy compared with other conditions with peripheral (ie, unilateral nerve damage) or central lesions of the somatosensory system, with 42% of patients having abnormal increased frequency of PHS compared with healthy individuals. The QST profiles were different for patients with polyneuropathy compared with patients with other neuropathies. Patients with polyneuropathy showed on average a general sensory loss on all sensory parameters, whereas the other groups also exhibited sensory gain, for example, lower pressure pain thresholds. Interestingly, we found no differences between QST profiles of patients with polyneuropathy with and without PHS. This result is in contrast to previous findings indicating that patients with uremic polyneuropathy experiencing PHS also exhibited less cold sensitivity,^40^ and patients with chemotherapy-induced neuropathy experiencing PHS had less warm sensitivity.^32^ These findings were not reproduced in our population, which covers polyneuropathy of diabetic, HIV, idiopathic, chemotherapy-induced, but not uremic aetiology. A previous study on diabetic children also did not find an association of PHS with sensory loss to cooling.^4^ These differences across studies may be related to symptoms severity. In our data, all patients with polyneuropathy already had major thermosensory deficits, suggesting potentially more severe polyneuropathy than in the patient populations investigated in other studies.

### 4.2. Paradoxical heat sensation is less frequent and related to thermal sensory loss in patients with unilateral nerve damage and central lesions

In patients with unilateral nerve damage or central lesions, PHS was similarly frequent across groups and associated with thermal detection deficits. This result suggests that PHS reflects loss of both C-fibre and Aδ-fibre function or their associated pathways in the central nervous system, although the relative importance of individual pathways cannot be determined from this study because the nervous lesions in these patients are rarely selective for single thermal pathways. Thus, the prediction from experimental A-fibre block studies in healthy individuals^11,38,39^ that selective loss of cold detection is linked to PHS is difficult to assess in this study. However, our finding that PHS is related to functional losses in thermal, but not mechanical, modalities suggests that PHS reflects a specific deficit in the function of small fibres involved in thermosensation, rather than a general sensory deficit.

### 4.3. Association between paradoxical heat sensation and Neuropathic Pain Symptom Inventory ratings

In the NPSI ratings, the presence of burning pain was similar in frequency in patients with and without PHS. Burning sensations are believed to be associated with loss of small fibre function^17^ and to be a result of a disinhibition of warm detection neurons caused by rarefication of cold-related fibres.^7^ In our analysis, spontaneous burning sensation reported by patients was lower for patients reporting PHS. Furthermore, PHS was associated with lower intensity of electric shock–like pain, which is considered to reflect small, myelinated fibre-related pain. These data suggest that impaired or unimpaired function of Aδ fibres and C fibres is relevant.

### 4.4. Limitations

In this study, PHS was assessed during 6 alternating warming and cooling stimuli, and in most cases, a single warm report during cooling of the skin was sufficient to classify the patient as reporting PHS. This has been a standard approach in the field in the past 20 years.^3,4,14,16,19–21,25,27^ However, other methods to assess PHS have been described previously. Yosipovitch and colleagues applied a series of 8 alternating warming and cooling stimuli and considered patients reporting PHS as those individuals who reported at least 3 or 4 warming sensations during cooling of the skin.^40^ By contrast, in our protocol, patients reporting PHS were patients who experienced 1 or 2 of 3 warm sensations during cooling.

Another limitation was the selection of the body site where QST was performed. This selection was not standardised for most patients' aetiologies, except for polyneuropathy. The body region for QST assessment was selected based on where the patient reported the most prominent pain or strongest somatosensory dysfunction in case of painless conditions. Consequently, while the tested body region could have been anywhere on the body, the reference values are only defined for hand, feet, trunk, and face. For each patient, we used reference values that were the closest approximation to the tested area. Finally, temporal and spatial summation effects may be underestimated^1^ because the battery of thermal and mechanical measures included in the QST according to the DFNS protocol only captures short-term effects (ie, each measure is obtained within a limited amount of time), rather than long-term effects, which may be more closely related to spontaneous pain symptoms.

## 5. Conclusions

We found that PHS was common not only in patients with polyneuropathy but also in patients with central lesions or unilateral peripheral injury. In patients with central or unilateral peripheral lesions, the presence of PHS was associated with reduced thermosensitive small fibre function, most likely including thinly myelinated Aδ fibres and unmyelinated C fibres. Our findings suggest that PHS is not a general sign of nerve damage, but rather a thermosensory-specific small fibre dysfunction, the mechanisms of which include a disinhibition phenomenon. In healthy subjects, the thermoreceptive and nociceptive circuitries include a pathway by which mild cooling can induce a percept of warmth or heat, and this circuitry is normally suppressed by thermosensory small fibre pathways. By contrast, our patient data indicate that the association between the presence of elevated PHS and lower NPSI scores for electric shock–like and burning pain quality plays an important role for Aδ than C fibres. Mechanistically, the inhibitory interactions between different small fibre pathways deserve more experimental studies; our findings suggest that there likely are additional inhibitory mechanisms distinct from the known gate control or the brainstem descending modulatory system.

Paradoxical heat sensation is an interesting sensory phenomenon that can be easily detected and is reported spontaneously by patients. This makes it an interesting parameter for clinical assessment of neuropathy. We argue that PHS should be included in the list of sensory signs of small fibre functional sensory loss,^15^ although at face value it seems to be a positive phenomenon. It may be an easy way to clinically screen for loss of small fibre function with a dichotomous outcome (PHS yes or no) and more sensitive than assessing abnormal thermal detection thresholds using DFNS reference values.

## Conflict of interest statement

The authors declare that they have no competing interests.
